# The association between protease inhibitors and anal cancer outcomes in veterans living with HIV treated with definitive chemoradiation: a retrospective study

**DOI:** 10.1186/s12885-021-08514-z

**Published:** 2021-07-05

**Authors:** Alison K. Yoder, David S. Lakomy, Yongquan Dong, Suchismita Raychaudhury, Kathryn Royse, Christine Hartman, Peter Richardson, Donna L. White, Jennifer R. Kramer, Lilie L. Lin, Elizabeth Chiao

**Affiliations:** 1 University of Texas Health Science Center at Houston, McGovern School of Medicine, Houston, TX USA; 2 Department of Radiation Oncology, The University of Texas MD Anderson Cancer Center, Houston, TX USA; 3 Dartmouth College Geisel School of Medicine, Hanover, NH USA; 4 Department of Medicine, Baylor College of Medicine, 1155 Pressler St. Unit, Houston, 1340 USA; 5 Michael E. DeBakey Veterans Affairs Medical Center, Houston, TX USA

**Keywords:** HIV, Protease inhibitors, Anal carcinoma, Squamous cell carcinoma, Chemoradiation

## Abstract

**Background:**

The incidence of anal squamous cell carcinoma has been increasing, particularly in people living with HIV (PLWH). There is concern that radiosensitizing drugs, such as protease inhibitors, commonly used in the management of HIV, may increase toxicities in patients undergoing chemoradiation. This study examines treatment outcomes and toxicities in PLWH managed with and without protease inhibitors who are receiving chemoradiation for anal cancer.

**Methods:**

Patient demographic, HIV management, and cancer treatment information were extracted from multiple Veterans Affairs databases. Patients were also manually chart reviewed. Among PLWH undergoing chemoradiation for anal carcinoma, therapy outcomes and toxicities were compared between those treated with and without protease inhibitors at time of cancer treatment. Statistical analysis was performed using chi-square, Cox regression analysis, and logistic regression.

**Results:**

A total of 219 PLWH taking anti-retroviral therapy undergoing chemoradiation for anal cancer were identified and included in the final analysis. The use of protease inhibitors was not associated with any survival outcome including colostomy-free survival, progression-free survival, or overall survival (all adjusted hazard ratio *p*-values> 0.05). Regarding toxicity, protease inhibitor use was not associated with an increased odds of hospitalizations or non-hematologic toxicities; however, protease inhibitor use was associated with increased hospitalizations for hematologic toxicities, including febrile neutropenia (*p* < 0.01).

**Conclusion:**

The use of protease inhibitors during chemoradiation for anal carcinoma was not associated with any clinical outcome or increase in non-hematologic toxicity. Their use was associated with increased hospitalizations for hematologic toxicities. Further prospective research is needed to evaluate the safety and efficacy of protease inhibitors for patients undergoing chemoradiation.

## Background

Cancers of the anus constitute approximately 8300 new cases and 1280 deaths per year in the United States [1]. While anal cancer remains a relatively rare malignancy, its incidence has steadily risen [2]. In particular, the introduction of highly active antiretroviral therapy (ART) and the subsequent shift of HIV into a chronic condition has played an important role in this increase [2]. As several studies have shown, HIV-infection with ART therapy is associated with both an increase in the risk of anal cancer as well as higher relapse rates following treatment [3–5].

Chemoradiation (CRT) is the mainstay of treatment for anal cancer that has any nodal involvement or is greater than T1 (and select T2) at diagnosis [6]. Radiation typically consists of a dose of 50–54 Gy to gross disease [7]. While several chemotherapy regimens can be utilized, the most widely used consists of 5-flurouracil (5-FU) and mitomycin C [8], however, 5-flurouracil and cisplatin [9] or capecitabine with mitomycin [10] are acceptable alternatives per National Comprehensive Cancer Network (NCCN) guidelines [11]. Regardless of regimen used, however, toxicity concerns abound. The most common toxicities consist of hematologic, dermatologic, gastrointestinal, and genitourinary complications [7–10]. These toxicities are often so severe that therapy completion rates remains a challenge as many trials have shown significant gaps, delays, or inability to complete full chemotherapy regimens [12].

Thus, there are concerns that drugs that serve as radiosensitizers might be difficult to tolerate during radiation treatment, possibly rendering them contraindicated. One auspicious class of radiosensitizing agents includes protease inhibitors (PIs) targeted against HIV-1. PIs have demonstrated in vitro and in vivo radiosensitizing effects in a variety of tumor cell lines [13–16]. Due to the promising preclinical data, several phase I studies testing the HIV-1 protease inhibitor nelfinavir combined with chemoradiation have been conducted for multiple cancer sites with promising results [17–24]. In one study of concurrent nelfinavir with CRT for non-small cell lung cancer [18], for example, they saw a median survival of 41.1 months, which was beyond historic norms for standard CRT of 17.0 months [25]. While treatment outcomes for PI results are promising, the question remains as to whether the radiosensitization from PIs, which lends to its increased efficacy, may also result in enhanced toxicity of normal tissue. Although randomized clinical trials would be the optimal avenue through which to study PI radiosensitization, people living with HIV (PLWH) are a natural cohort of patients who regularly take these medications during treatment for anal cancer. In this study we retrospectively analyze data from the Department of Veterans Affairs to examine the effect of PI usage during CRT for anal squamous cell carcinoma on survival and toxicities.

## Methods

### Patient selection and characteristics

Patient information and data was extracted from the United States Department of Veterans Affairs (VA) databases and registries accessible through the VA Informatics and computing infrastructure (VINCI), including the VA Corporate Data Warehouse (CDW) and the VA Central Cancer Registry (CCR) from years 2000 to 2016. The CDW is an automated VA database that includes all patient data including inpatient and outpatient encounters, laboratory information, and pharmacy information. It is updated in near real-time and is available for all VA users. The VA CCR is a national data repository for over 750,000 VA patients with cancer [26, 27]. Each VA medical center has its own cancer registrar who manually extracts the data, which is then aggregated into a national cancer registry. Data was abstracted from both databases. VINCI was then used to manually chart review the data pulled from CDW and CCR as well as to extract additional variables not available in the databases.

**Fig. 1:**
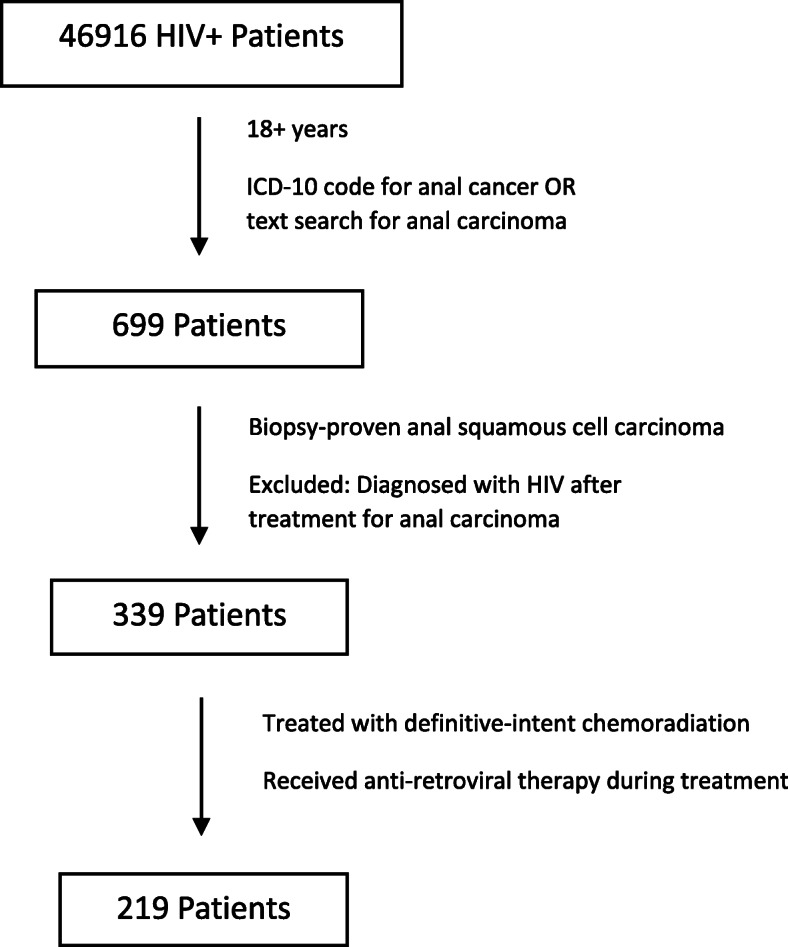
Patient Selection

Inclusion criteria for patients included veterans with HIV-positivity, biopsy-proven anal squamous cell carcinoma, at least 18 years of age at start of treatment, definitive CRT treatment, and on ART during treatment. HIV-infection was determined as “positive” as described by Kramer et al. [28], whereby patients must fulfill two of the three following criteria: (1) at least one positive HIV antibody test by ELISA or Western Blot, viral load (any ± interderminate), or tested for CD4+ count; (2) at least one inpatient or outpatient pharmacy records for HIV ART medication; and (3) *International Classification of Diseases, Ninth Revision (ICD-9)* (042 or V08) or *ICD-10* code (B20 or Z21) for HIV. Anal cancer was identified via primary site and histology codes 1540–1548, 2304–2306, C19–20, C210–212, D011–013 as well as text searches; biopsy-proven confirmation was determined through chart review. The type of ART medication classified as PI vs. non-PI based was based on the medication the patient was taking at the start of chemoradiation. Exclusion criteria included patients who were diagnosed with HIV after CRT for anal cancer, patients treated with palliative intent, and those who were treated with primary surgery (see Fig. 1).

Patient information collected included patient characteristics such as age at diagnosis, race/ethnicity, body mass index (BMI), smoking and alcohol use, history of prior cancers; disease stage at diagnosis; HIV-related variables including any changes in type of ART used within 90 days of starting treatment, CD4 at diagnosis and nadir CD4 during radiation; treatment variables including radiation dose and modality, duration, and completion, and chemotherapy regimen including if there was dose reduction; outcome data including recurrence date and site, receipt of colostomy and date, and vital status as of May 2019; and toxicity information including acute and chronic toxicities as well as risk of hospitalization. Toxicities were based on the National Cancer Institute’s Common Terminology Criteria for Adverse Events Version 5.0.

### Statistical analysis

We performed chi-square and Fisher’s exact analysis to compare sociodemographic risk factors, clinical characteristics and outcomes among PLWH who received PIs during treatment to those who did not (i.e., non-PI users). Kaplan-Meier curves were constructed, and log-rank tests were performed to compare survival curves for PI users and non-PI users for overall survival (OS), recurrence-free survival (RFS) and colostomy-free survival (CFS). Cox proportional hazards (PH) analysis was performed to assess for differences in RFS, CFS, and OS, respectively, in PI users and non-users. We interrogated all variables that on univariate analysis had an overall *p*-value or individual stratum specific Wald test *p*-value for non-binary variables of < 0.25 to allow for potential heterogeneity as well as race/ethnicity and age regardless of significance in multivariable analyses. We employed backward stepwise selection to final reported multivariable models for OS, RFS, and CFS in PI users and non-users with other covariates retained in final reported model if overall or stratum-specific *p*-value < 0.10, and with race/ethnicity and age forced in final model regardless of significance given their known strong association with cancer risk and to aid comparison with other studies. We employed this approach in multivariable analyses to maximize precision of effect estimates for our primary variable of interest, PI usage vs. non-usage, and to minimize risk of overfitting while also adjusting for race/ethnicity and age given they are well-established risk factors for most cancers and any other strongly associated covariates. Effect estimates are reported as hazard ratios (HR) with associated 95% confidence intervals (CIs) and *p*-values, with two-sided *p*-values less than < 0.05 considered significant and *p*-values between 0.05 and 0.10 considered approaching significance.

To analyze toxicity, logistic regression analysis was performed to analyze variables associated with ≥ grade 3 acute and late toxicities as well as hospitalizations. Acute toxicities were defined as occurring within 90 days of starting treatment; late toxicities were defined as occurring at least 90 days after starting treatment until the end of follow-up. Hospitalizations were included in analysis if they occurred during treatment or within 90 days of treatment onset. Our logistic regression modeling approach was the same as described for Cox models above including use of the same criteria in univariable analyses followed by multivariable analysis. We also employed logistic regression to examine the association between the use of PIs and relative risk of specific treatment related outcomes as well as of all specific individual toxicities. However, given the even further limited sample size for analyses evaluating specific individual toxicities, our primary analyses for them is our univariable analysis. For purposes of hypothesis generation we also performed exploratory multivariable analyses for specific treatment and toxicity related outcomes significant at *p* < 0.05 given further reduction in study power. Logistic regression-based model results are presented as odds ratios with associated 95% confidence intervals and *p*-values, with two-sided *p*-values < 0.05 considered statistically significant. SAS v9.4 was used for analysis.

## Results

### Patient and HIV characteristics

A total of 339 veteran patients were found to be HIV-positive and diagnosed with anal squamous cell carcinoma. Of these, 219 PLWH were treated with definitive-intent CRT and received at least one anti-retroviral drug during their treatment and were included in the final analysis (Table 1). Median age at time of HIV diagnosis was 46 years (interquartile range (IQR) 40–53) and median age at time of anal cancer diagnosis was 53.7 years (IQR 48.9–60.8). Median follow up was 4.7 years (IQR 2.1–8.6). The majority of patients were Caucasian (50.7%, *n* = 111), and nearly half were stage II at diagnosis (48.9%, *n* = 107). Most patients had smoked cigarettes at some point during their lifetime (68.9%, *n* = 151), and 51.6% (*n* = 113) still smoked at the time of cancer diagnosis. There was no significant difference in age, race/ethnicity, alcohol use, or smoking status between PLWH who were treated with PIs and those who were not (*p* = 0.19, 0.78, 0.73, and 0.80 respectively). PLWH treated with PIs were significantly less likely to be obese; only 9.2% had a BMI at time of treatment of 30 or greater, compared to 16.7% of PLWH who were not treated with PIs (*p* = 0.05).

**Table 1:** Demographic and treatment variables during chemoradiation

	Taking protease inhibitors n (%)	Not taking protease inhibitors n (%)	***p***-value
	153 (69.86)	66 (30.14)	
**Age at diagnosis**			0.19
< 40	10 (6.54)	2 (3.03)	
40–59	104 (67.97)	40 (60.61)	
60+	39 (25.49)	24 (36.36)	
**Race/Ethnicity**			0.78
African American	55 (35.95)	21 (31.82)	
Caucasian	78 (50.98)	33 (50.00)	
Hispanic	11 (7.19)	7 (10.61)	
Other/Unknown	9 (5.88)	5 (7.58)	
**Year HIV Diagnosed**			0.37
< 2000	103 (67.32)	39 (59.09)	
2000–2009	39 (25.49)	23 (34.85)	
2010–2016	11 (7.19)	4 (6.06)	
**Smoker**			0.80
Current	76 (49.67)	37 (56.06)	
Former	28 (18.30)	10 (15.15)	
Never	41 (26.80)	15 (22.73)	
Unknown	8 (5.23)	4 (6.06)	
**Stage**			0.28
I	21 (13.73)	15 (22.73)	
II	73 (47.71)	34 (51.52)	
IIIA	15 (9.80)	3 (4.55)	
IIIB	35 (22.88)	13 (19.70)	
IV	6 (3.92)	1 (1.52)	
Unknown	3 (1.96)	0 (0)	
**CD4 at HIV Diagnosis**			< 0.01
≤ 200	55 (35.95)	20 (30.30)	
> 200	59 (38.56)	42 (63.64)	
Unknown	39 (25.49)	4 (6.06)	
**CD4 at Cancer Diagnosis**			< 0.01
≤ 200	43 (28.10)	8 (12.12)	
> 200	101 (66.01)	58 (87.88)	
Unknown	9 (5.88)	0 (0)	
**Nadir CD4 during Radiation**			< 0.01
≤ 200	79 (51.63)	20 (30.30)	
> 200	33 (21.57)	25 (37.88)	
missing	41 (26.80)	21 (31.82)	
**BMI**			0.05
< 25	76 (49.67)	37 (56.06)	
25–29.9	53 (34.64)	18 (27.27)	
≥ 30	14 (9.15)	11 (16.67)	
Unknown	10 (6.54)	0 (0)	
**Alcohol Use**			0.73
Yes	57 (37.25)	23 (34.85)	
No	96 (62.75)	43 (65.15)	
**History of prior cancer**			0.14
Yes	22 (14.38)	14 (21.21)	
No	129 (84.31)	49 (74.24)	
Unknown	2 (1.31)	3 (4.55)	
**Radiation Modality**			0.87
2D + 3D	54 (35.29)	21 (31.82)	
IMRT	52 (33.99)	23 (34.85)	
Unknown	47 (30.72)	22 (33.33)	
**Completed Radiation Treatment**			0.48
Yes	134 (87.58)	60 (90.91)	
No	19 (12.42)	6 (9.09)	
**Length of Radiation Treatment**			0.59
≤ 50 days	86 (56.21)	42 (63.64)	
> 50 days	61 (39.87)	22 (33.33)	
Unknown	6 (3.92)	2 (3.03)	
**Chemotherapy Regimen**			0.05
5FU + mitomycin C	115 (75.16)	47 (71.21)	
5FU + cisplatin	20 (13.07)	4 (6.06)	
other	18 (11.76)	15 (22.73)	
**Chemotherapy Dose Reduction**			0.25
Yes	49 (32.03)	16 (24.24)	
No	104 (67.97)	50 (75.76)	

Most patients had CD4 count greater than 200 (cells/mL) at time of cancer diagnosis (72.6%, *n* = 159). The majority of patients were on PIs at time of treatment, (69.9%, *n* = 153). PLWH treated with PIs were less likely to have CD4 count greater than 200 at time of HIV diagnosis (38.6%, *n* = 59 vs 63.6%, *n* = 42; *p* < 0.01) or at time of cancer diagnosis (66.0%, *n* = 101 vs 87.9%, *n* = 58; p < 0.01). Twenty-five patients changed ART medications in the 90 days following the start of radiation treatment. An additional sensitivity analysis was done limiting the cohort to only the patients who did not change the type of medication they were taking, which showed no significant difference between the two cohorts in either univariate or multivariable analysis (data not shown).

### Chemoradiation treatment characteristics and outcome analysis

All patients included in this study were treated with curative-intent CRT, though 25 patients (11.4%) did not complete all fractions of radiation. There was no significant difference in radiation completion between those who received PIs and those who did not (*p* = 0.48). Seventy-five PLWH (34.2%) received intensity-modulated radiation therapy (IMRT), 75 (34.2%) received 2D or 3D radiation, and 69 (31.5%) did not have this information available. The median radiation dose was 54 Gy. 5-FU and mitomycin was the most common chemotherapy regimen with 162 patients (74.0%) receiving this treatment. Patients who were on PIs were more likely to receive 5-FU and cisplatin than patients who were not taking PIs (13.1%, *n* = 20 vs 6.1%, *n* = 4; *p* = 0.05). A significant percentage of patients required a chemotherapy dose reduction (29.7%, *n* = 65) during their treatment.

**Fig. 2:**
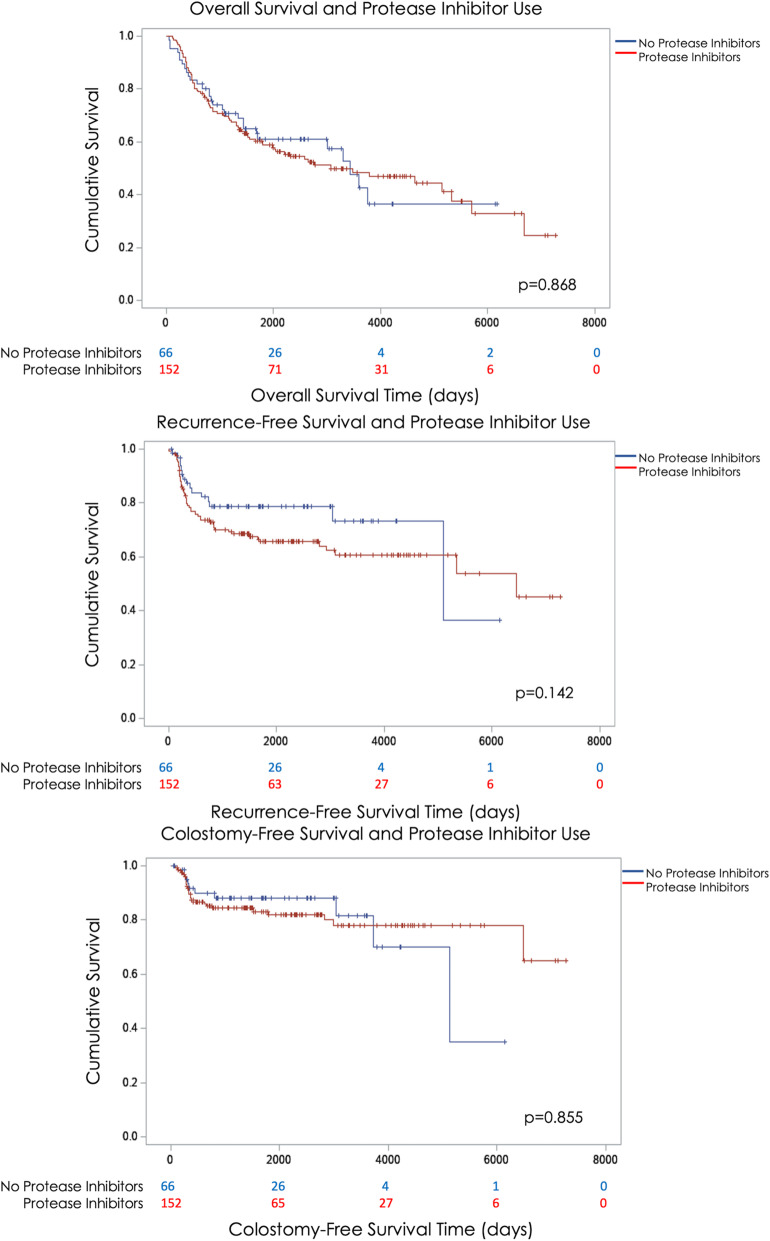
Kaplan-Meier Curves of overall survival, recurrence-free survival, and colostomy-free survival of PLWH undergoing definitive chemoradiation for anal cancer who took protease inhibitors compared to those who did not

There was no significant difference in any outcome measure between PLWH who took PIs and those that did not (Fig. 2). On univariate Cox analysis, there were no significant differences with OS, RFS, or CFS in non-users of PIs when compared to patients who did receive PIs (HR 1.04 [0.68–1.59], 1.53 [0.86–2.72], 1.07 [0.52–2.23], respectively; all *p* > 0.05) (Table 2). Increasing stage was associated with both worse OS as well as increased chance of tumor recurrence (OS HR 2.38 [1.17–4.82], 2.94 [1.16–7.41], 3.07 [1.44–6.53], and 3.83 [1.18–12.47] for stage II, IIIA, IIIB, and IV, respectively; all *p* < 0.05). Race/ethnicity, age, and alcohol use were not associated with OS, RFS, or CFS (all *p* > 0.5).

**Table 2:** Cox regression models for overall survival, recurrence-free-survival, and colostomy-free survival for patients living with HIV treated with definitive chemoradiation

	Overall survival (OS)				Recurrence-free Survival (RFS)				Colostomy-free Survival (CFS)			
	Univariate Model		Adjusted Model		Univariate Model		Adjusted Model		Univariate Model		Adjusted Model	
	HR (95%CI)	***p***-value	HR (95%CI)	***p***-value	HR (95%CI)	***p***-value	HR (95%CI)	***p***-value	HR (95%CI)	***p***-value	HR (95%CI)	***p***-value
**Age at diagnosis**												
< 40	1.00	Ref	1.00	Ref	1.00	Ref	1.00	Ref	1.00	Ref	1.00	Ref
40–59	0.78 (0.37–1.64)	0.51	0.69 (0.29–1.60)	0.39	0.65 (0.29–1.47)	0.30	0.66 (0.28–1.54)	0.34	0.99 (0.29–3.41)	0.98	0.94 (0.26–3.34)	0.92
60+	1.44 (0.66–3.17)	0.36	1.65 (0.67–4.05)	0.28	0.60 (0.24–1.51)	0.28	0.53 (0.20–1.43)	0.21	0.87 (0.22–3.48)	0.85	0.79 (0.19–3.23)	0.74
**Race/Ethnicity**												
Caucasian	1.00	Ref	1.00	Ref	1.00	Ref	1.00	Ref	1.00	Ref	1.00	Ref
African American	1.25 (0.82–1.89)	0.29	1.31 (0.83–2.06)	0.25	1.32 (0.79–2.18)	0.29	1.17 (0.67–2.04)	0.57	0.98 (0.47–2.01)	0.95	1.06 (0.50–2.27)	0.87
Hispanic	0.68 (0.29–1.60)	0.38	0.69 (0.27–1.81)	0.45	0.71 (0.25–2.02)	0.52	0.65 (0.22–1.94)	0.44	0.99 (0.29–3.35)	0.99	1.17 (0.33–4.12)	0.81
Oher/Unknown	1.64 (0.80–3.33)	0.18	2.10 (0.93–4.74)	0.07	0.98 (0.35–2.77)	0.97	1.36 (0.46–4.06)	0.58	1.39 (0.41–4.71)	0.60	1.22 (0.35–4.24)	0.75
**HIV Medication**												
No Protease Inhibitor	1.00	Ref	1.00	Ref	1.00	Ref	1.00	Ref	1.00	Ref	1.00	Ref
Protease Inhibitor	1.04 (0.68–1.59)	0.87	0.86 (0.53–1.38)	0.53	1.53 (0.86–2.72)	0.15	1.34 (0.74–2.43)	0.33	1.07 (0.52–2.23)	0.85	1.03 (0.49–2.15)	0.94
**Year HIV Diagnosed**												
< 2000	1.00	Ref			1.00	Ref			1.00	Ref		
2000–2009	0.77 (0.48–1.22)	0.26			0.82 (0.47–1.43)	0.48			1.22 (0.61–2.45)	0.58		
2010–2016	0.98 (0.42–2.27)	0.96			0.63 (0.19–2.01)	0.43			–	–		
**Smoker**												
Never	1.00	Ref	1.00	Ref	1.00	Ref			1.00	Ref		
Former	1.76 (0.96–3.22)	0.07	1.65 (0.82–3.29)	0.16	2.01 (1.01–4.00)	0.05			1.14 (0.47–2.77)	0.77		
Current	1.45 (0.88–2.40)	0.15	1.51 (0.83–2.77)	0.18	1.12 (0.62–2.04)	0.71			0.60 (0.28–1.27)	0.18		
Unknown	2.54 (1.16–5.55)	0.02	4.08 (1.71–9.73)	< 0.01	0.97 (0.28–3.35)	0.97			0.84 (0.19–3.73)	0.82		
**Stage**												
I	1.00	Ref	1.00	Ref	1.00	Ref	1.00	Ref	1.00	Ref		
II	2.38 (1.17–4.82)	0.02	3.16 (1.50–6.62)	< 0.01	1.75 (0.77–3.97)	0.18	1.77 (0.75–4.16)	0.19	2.06 (0.70–6.00)	0.19		
IIIA	2.94 (1.16–7.41)	0.02	4.63 (1.67–12.82)	< 0.01	1.88 (0.60–5.93)	0.28	2.03 (0.62–6.62)	0.24	2.71 (0.68–10.86)	0.16		
IIIB	3.07 (1.44–6.53)	< 0.01	3.16 (1.42–7.06)	< 0.01	3.10 (1.32–7.31)	< 0.01	3.01 (1.20–7.55)	0.02	1.98 (0.59–6.59)	0.27		
IV	3.83 (1.18–12.47)	0.03	2.41 (0.69–8.42)	0.17	5.73 (1.67–19.6)	< 0.01	6.14 (1.68–22.44)	< 0.01	–	–		
Unknown	3.66 (0.79–16.99)	0.10	0.94 (0.17–5.11)	0.94	–	–	–	–	–	–		
**CD4 at Cancer Diagnosis**												
≤ 200	1.00	Ref			1.00	Ref			1.00	Ref		
> 200	0.90 (0.58–1.41)	0.66			0.63 (0.38–1.06)	0.08			1.09 (0.49–2.42)	0.83		
Unknown	1.00 (0.41–2.47)	0.99			0.59 (0.17–2.01)	0.40			1.78 (0.46–6.93)	0.40		
**Nadir CD4 during Radiation**												
≤ 200	1.00	Ref			1.00	Ref			1.00	Ref		
> 200	0.74 (0.45–1.22)	0.24			0.75 (0.42–1.33)	0.32			0.88 (0.40–1.90)	0.74		
Unknown	2.54 (1.16–5.55)	0.76			0.64 (0.35–1.15)	0.13			0.73 (0.33–1.62)	0.43		
**BMI**												
< 25	1.00	Ref	1.00	Ref	1.00	Ref	1.00	Ref	1.00	Ref		
25–29.9	0.72 (0.47–1.11)	0.14	0.49 (0.30–0.81)	< 0.01	0.84 (0.50–1.42)	0.51	0.62 (0.34–1.11)	0.11	1.09 (0.55–2.18)	0.81		
≥ 30	0.39 (0.17–0.91)	0.03	0.61 (0.25–1.51)	0.29	0.30 (0.09–0.95)	0.04	0.32 (0.10–1.06)	0.06	0.22 (0.03–1.61)	0.14		
Unknown	1.09 (0.51–2.32)	0.82	0.76 (0.32–1.77)	0.52	0.88 (0.31–2.52)	0.82	0.71 (0.24–2.13)	0.54	1.35 (0.39–4.72)	0.63		
**Alcohol Use**												
No	1.00	Ref			1.00	Ref			1.00	Ref		
Yes	0.93 (0.63–1.40)	0.74			0.69 (0.41–1.16)	0.16			0.85 (0.43–1.70)	0.65		
**History of prior cancer**												
No	1.00	Ref			1.00	Ref			1.00	Ref		
Yes	0.87 (0.51–1.48)	0.60			0.71 (0.35–1.43)	0.33			0.91 (0.38–2.18)	0.83		
**Radiation Modality**												
2D + 3D	1.00	Ref	1.00	Ref	1.00	Ref			1.00	Ref		
IMRT	0.65 (0.40–1.06)	0.08	0.50 (0.29–0.86)	0.01	0.70 (0.39–1.26)	0.23			0.59 (0.25–1.39)	0.22		
Unknown	0.72 (0.46–1.13)	0.15	0.60 (0.36–0.98)	0.04	0.78 (0.45–1.37)	0.39			0.99 (0.48–2.05)	0.97		
**Completed Radiation Treatment**												
No	1.00	Ref	1.00	Ref	1.00	Ref	1.00	Ref	1.00	Ref		
Yes	0.40 (0.23–0.69)	< 0.01	0.26 (0.14–0.50)	< 0.01	0.61 (0.30–1.24)	0.17	0.44 (0.20–0.95)	0.04	1.56 (0.37–6.51)	0.54		
**Length of Radiation Treatment**												
≤ 50 days	1.00	Ref			1.00	Ref	1.00	Ref	1.00	Ref	1.00	Ref
> 50 days	1.67 (1.13–2.47)	0.01			1.48 (0.91–2.43)	0.12	1.14 (0.66–1.97)	0.63	1.55 (0.77–3.10)	0.22	1.56 (0.77–3.17)	0.22
Unknown	2.54 (1.16–5.55)	0.15			2.87 (1.11–7.39)	0.03	3.63 (1.32–9.99)	0.01	5.34 (1.94–14.71)	< 0.01	5.48 (1.93–15.55)	< 0.01
**Chemotherapy Regimen**												
5FU + mitomycin C	1.00	Ref			1.00	Ref			1.00	Ref		
5FU + cisplatin	1.39 (0.81–2.39)	0.23			0.84 (0.38–1.84)	0.66			1.91 (0.82–4.46)	0.13		
Other	1.07 (0.63–1.85)	0.80			0.92 (0.47–1.81)	0.81			1.44 (0.62–3.37)	0.40		
**Chemotherapy Dose Reduction**												
No	1.00	Ref			1.00	Ref			1.00	Ref		
Yes	1.40 (0.94–2.08)	0.10			1.46 (0.90–2.39)	0.13			1.23 (0.62–2.45)	0.56		
**Hospitalization during treatment**												
No	1.00	Ref	1.00	Ref	1.00	Ref			1.00	Ref		
Yes	2.05 (1.40–3.00)	< 0.01	1.92 (1.24–2.96)	< 0.01	2.05 (1.28–3.30)	< 0.01			1.37 (0.71–2.64)	0.35		

In the multivariable analysis, the use of PIs continued to have no significant association with OS, RFS, or CFS (HRadjusted (adj) 0.86 [0.53–1.38], 1.34 [0.74–2.43], 1.03 [0.49–2.15], respectively; all *p* > 0.05). Increasing stage remained significantly associated with both OS and RFS; however, stage was not associated with CFS and therefore it was not retained in the final model. Neither race nor age were significantly associated with OS, RFS, or CFS. PLWH with a BMI between 25 and 29.9, or clinically overweight, had improved OS than those who with BMI < 25 or normal weight (HRadj 0.49 [0.30–0.81], *p* < 0.01). In addition, those treated with IMRT had improved OS than those treated with either 2D or 3D radiation (HRadj 0.50 [0.29–0.86], *p* = 0.01).

Patients who completed radiation treatment had significantly improved OS and RFS than those that did not in multivariable analysis (both *p* < 0.05). There was no association between PLWH who took PIs and their ability to complete radiation treatment (*p* = 0.48), or the length of radiation treatment (*p* = 0.33) (Table 4). PLWH who required a dose reduction of chemotherapy had decreased OS and RFS, though these were not statistically significant findings (HR 1.40 [0.94–2.08], 1.46 [0.90–2.39]; respectively) (Table 2). There was no association between the use of PIs and patients requiring dose reductions in chemotherapy (*p* = 0.25) (Table 4).

### Toxicity analysis

The use of PIs did not increase the risk for either acute or long-term non-hematologic toxicity in PLWH treated with definitive CRT for anal cancer in either univariate or multivariable logistic regression analysis (all *p* > 0.05, Table 3). However, patients on PI ART regimens had an increased risk of hospitalization (Odds ratio (OR) 2.44 [1.27–4.65], *p* < 0.01) compared to those who did not take PIs on univariate analysis. This association continued after controlling for confounding variables in the multivariable analysis (ORadj 2.17 [1.04–4.56], *p* = 0.04).

**Table 3:** Logistic regression models for grade 3 and higher acute non-hematologic toxicities, late non-hematologic toxicities, and hospitalizations for patients living with HIV undergoing definitive chemoradiation for anal carcinoma

	Acute ***≥*** G3 non-hematologic toxicities				Late ***≥*** G3 non-hematologic toxicities				Hospitalization			
	Univariate Model		Adjusted Model		Univariate Model		Adjusted Model		Univariate Model		Adjusted Model	
	OR (95%CI)	p-value	OR (95%CI)	***p***-value	OR (95%CI)	***p***-value	OR (95%CI)	***p***-value	OR (95%CI)	***p***-value	OR (95%CI)	***p***-value
**Age at diagnosis**												
< 40	1.00	Ref	1.00	Ref	1.00	Ref	1.00	Ref	1.00	Ref	1.00	Ref
40–59	0.40 (0.12–1.39)	0.15	0.44 (0.12–1.62)	0.22	0.46 (0.09–2.32)	0.34	0.43 (0.08–2.26)	0.32	0.60 (0.18–1.95)	0.40	0.49 (0.13–1.88)	0.30
60+	0.55 (0.15–2.01)	0.37	0.58 (0.15–2.26)	0.43	1.30 (0.25–6.68)	0.75	1.42 (0.27–7.60)	0.68	0.58 (0.17–1.99)	0.38	0.61 (0.15–2.50)	0.49
**Race/Ethnicity**												
Caucasian	1.00	Ref	1.00	Ref	1.00	Ref	1.00	Ref	1.00	Ref	1.00	Ref
African American	0.63 (0.35–1.14)	0.12	0.63 (0.34–1.18)	0.15	1.22 (0.48–3.11)	0.67	1.49 (0.57–3.94)	0.42	1.71 (0.94–3.11)	0.08	1.80 (0.90–3.59)	0.10
Hispanic	1.14 (0.42–3.11)	0.79	1.18 (0.41–3.43)	0.76	1.82 (0.45–7.28)	0.40	1.87 (0.45–7.81)	0.39	1.00 (0.35–2.88)	0.99	0.97 (0.29–3.22)	0.96
Other/ Unknown	0.69 (0.22–2.11)	0.51	0.77 (0.14–2.48)	0.66	3.64 (0.98–13.56)	0.05	4.62 (1.17–18.22)	0.03	1.11 (0.35–3.55)	0.86	2.66 (0.68–10.31)	0.16
**HIV Medication**												
No Protease Inhibitor	1.00	Ref	1.00	Ref	1.00	Ref	1.00	Ref	1.00	Ref	1.00	Ref
Protease Inhibitor	1.38 (0.77–2.46)	0.28	1.34 (0.72–2.48)	0.36	1.27 (0.51–3.16)	0.61	1.55 (0.59–4.07)	0.37	2.44 (1.27–4.65)	< 0.01	2.17 (1.04–4.56)	0.04
**Year HIV Diagnosed**												
< 2000	1.00	Ref			1.00	Ref			1.00	Ref		
2000–2009	1.05 (0.58–1.91)	0.87			1.44 (0.59–3.49)	0.42			0.81 (0.43–1.51)	0.51		
2010–2016	1.28 (0.44–3.72)	0.65			2.12 (0.54–8.36)	0.28			1.38 (0.48–4.03)	0.55		
**Smoker**												
Never	1.00	Ref			1.00	Ref			1.00	Ref	1.00	Ref
Current	1.06 (0.56–2.00)	0.87			0.68 (0.24–1.89)	0.46			1.41 (0.71–2.80)	0.33	1.74 (0.76–4.01)	0.19
Former	0.90 (0.40–2.05)	0.80			1.87 (0.61–5.67)	0.27			2.83 (1.20–6.67)	0.02	3.21 (1.19–8.66)	0.02
Unknown	0.09 (0.01–0.75)	0.03			1.40 (0.25–7.76)	0.70			0.46 (0.09–2.32)	0.35	0.63 (0.11–3.62)	0.61
**Stage**												
I	1.00	Ref			1.00	Ref			1.00	Ref	1.00	Ref
II	1.37 (0.64–2.95)	0.41			2.77 (0.60–12.76)	0.19			1.32 (0.57–3.03)	0.52	1.14 (0.45–2.89)	0.78
IIIA	0.70 (0.21–2.29)	0.55			2.13 (0.27–16.48)	0.47			1.66 (0.50–5.47)	0.41	1.20 (0.32–4.52)	0.79
IIIB	2.14 (0.89–5.15)	0.09			2.43 (0.46–12.81)	0.30			2.60 (1.03–6.54)	0.04	2.14 (0.76–6.00)	0.15
IV	0.56 (0.10–3.28)	0.52			6.80 (0.77–59.75)	0.08			15.6 (1.66–146)	0.02	16.35 (1.47–181.9)	0.02
**Nadir CD4 during Radiation**												
≤ 200	1.00	Ref			1.00	Ref			1.00	Ref	1.00	Ref
> 200	0.59 (0.31–1.13)	0.11			0.77 (0.29–2.01)	0.59			0.48 (0.24–0.95)	0.03	0.53 (0.24–1.20)	0.13
Unknown	0.64 (.034–1.22)	0.18			0.49 (0.17–1.43)	0.19			0.40 (0.20–0.80)	< 0.01	0.38 (0.17–0.83)	0.02
**BMI**												
< 25	1.00	Ref	1.00	Ref	1.00	Ref			1.00	Ref	1.00	Ref
25–30	0.92 (0.51–1.67)	0.79	1.00 (0.54–1.86)	0.99	0.54 (0.22–1.36)	0.19			1.07 (0.58–1.95)	0.84	0.85 (0.43–1.70)	0.65
≥ 30	0.37 (0.14–0.95)	0.04	0.43 (0.16–1.14)	0.09	–	–			0.28 (0.09–0.86)	0.03	0.27 (0.07–0.95)	0.04
Unknown	0.95 (0.26–3.46)	0.94	0.88 (0.22–3.53)	0.85	0.55 (0.07–4.60)	0.58			0.62 (0.15–2.54)	0.51	0.75 (0.15–3.68)	0.72
**Radiation Modality**												
2D + 3D	1.00	Ref	1.00	Ref	1.00	Ref			1.00	Ref		
IMRT	0.58 (0.31–1.12)	0.10	0.67 (0.34–1.33)	0.26	0.78 (0.29–2.09)	0.62			0.72 (0.37–1.38)	0.32		
Unknown	0.45 (0.23–0.88)	0.02	0.49 (0.24–0.97)	0.04	0.98 (0.37–2.56)	0.96			0.64 (0.32–1.25)	0.19		
**Chemotherapy Regimen**												
5FU + mitomycin C	1.00	Ref			1.00	Ref			1.00	Ref		
5FU + cisplatin	0.62 (0.25–1.49)	0.28			1.60 (0.49–5.21)	0.44			1.09 (0.46–2.61)	0.84		
Other	0.97 (0.46–2.04)	0.93			1.43 (0.49–4.17)	0.51			0.57 (0.25–1.32)	0.19		

The majority of patients experienced grade 3 or higher hematologic toxicity (62.6%, *n* = 137). Patients who were taking PIs were significantly more likely to have any hematologic toxicity greater than grade 3 in univariable analysis (OR 2.12 [1.18–3.83], *p* = 0.01, Table 4). Specifically, they were more likely to experience at least grade 3 lymphopenia during treatment (OR 1.92 [1.05–3.51], *p* = 0.03). While grade 3 or greater leukopenia was more likely in patients on PI ART (OR 1.73 [0.93–3.23], *p* = 0.08), this was not a statistically significant finding. There was no difference in neutropenia, anemia, or thrombocytopenia in univariable analyses (all *p* > 0.05).

**Table 4:** Univariate Logistic Regression models for relative risk of select individual treatment and toxicity related outcomes according to PI use

Outcome	Taking a Protease Inhibitor Odds Ratio (95% CI)	***p***-value
**Treatment-related**		
Completed Radiation	0.71 (0.27–1.86)	0.48
Length of Radiation Treatment > 50 days	0.74 (0.40–1.36)	0.33
Required a Chemotherapy Dose Reduction	1.47 (0.76–2.84)	0.25
**Toxicities during treatment**		
Hospitalized for GI Toxicity	1.01 (0.25–4.02)	0.99
Hospitalized for Hematologic Toxicity	4.60 (1.56–13.5)	< 0.01
Hospitalized for Febrile Neutropenia	6.25 (1.44–27.2)	0.01
Hospitalized for Radiation Dermatitis	0.74 (0.21–2.63)	0.65
Acute Radiation Dermatitis at least Grade 3	0.94 (0.51–1.74)	0.85
Acute GI Toxicity at least Grade 3	1.94 (0.70–5.39)	0.20
Acute Oral Mucositis at least Grade 3	1.04 (0.35–3.07)	0.95
Late GI Toxicity at least Grade 3	1.56 (0.49–4.93)	0.45
Other Late Toxicity at least Grade 3	0.63 (0.17–2.32)	0.49
Any Acute Hematologic Toxicity	2.12 (1.18–3.83)	0.01
Acute Anemia at least Grade 3	1.47 (0.39–5.52)	0.57
Acute Leukopenia at least Grade 3	1.73 (0.93–3.23)	0.08
Acute Thrombocytopenia at least Grade 3	1.54 (0.71–3.34)	0.28
Acute Neutropenia at least Grade 3	1.73 (0.71–4.20)	0.23
Acute Lymphopenia at least Grade 3	1.92 (1.05–3.51)	0.03

PLWH on PI regimens were more likely to be hospitalized for febrile neutropenia or other hematologic toxicity than those who did not receive PIs (OR 6.25 [1.44–27.2], *p* = 0.01; OR 4.60 [1.56–13.5], *p* < 0.01, respectively). Only 3.5% of patients (*n* = 3) who were not on PIs at time of CRT were hospitalized for febrile neutropenia, and 7% (*n* = 6) were hospitalized for another hematologic toxicity. This is compared to 25 (16.3%) patients receiving PIs that were hospitalized for febrile neutropenia, and 35 (22.9%) that were hospitalized for any other hematologic toxicity. Hospitalizations during treatment was associated with decreased OS (HRadj 1.92 [1.24–2.96], *p* < 0.01) on multivariable analysis (Table 2). In exploratory multivariable models for the four treatment related toxicities significantly associated with PI use in univariable analysis, all remained similarly significantly associated in multivariable analysis (e.g., ORadj 5.25 95% CI (1.67–16.5) p < 0.01 for PI users compared to non-PI users for hospitalized for hematologic toxicity, data not shown).

There were no grade 3 or greater genitourinary complications seen in PLWH who did not receive PIs, and only two amongst those who did, a non-statistically significant difference. The use of PIs was not associated with any other specific acute or late non-hematologic toxicity on univariate analysis, including dermatitis, gastrointestinal complications, or oral mucositis (all *p* > 0.05) (Table 4).

## Discussion

In this retrospective study, we assessed survival outcomes and toxicity in anal cancer patients treated with definitive CRT in PLWH managed both with and without PI use. PI use was not significantly associated with any change in survival, recurrence, or need for a colostomy. In regard to toxicity, while PI use was associated with an increase in hospitalizations attributable to hematologic toxicities, including febrile neutropenia, no such association was found for other common toxicities including dermatologic or gastrointestinal complications. In addition, PI use was not associated with any chemotherapy or radiation treatment variable that was found to be associated with survival outcomes, such as the ability to complete radiation treatment.

Protease inhibitors, originally developed for HIV-treatment, have long shown anti-cancer effects. The etiology underlying this additional characterization is pleiotropic in nature. For one, PIs have shown direct cytotoxic effects including increased cell apoptosis due to increased endoplasmic reticulum stress and autophagy [29]. Additionally, for virally associated cancers, such as Human Papilloma Virus-induced anal and cervical cancer, PIs have been shown to target viral antigens that are necessary for viral replication, i.e. they have been observed to inhibit viral E6-mediated degradation of p53 [30]. Lastly, the most prominently researched anti-tumor mechanism is thought to be its downregulation of mitogenic growth signaling pathways, specifically PI3K/Akt [13, 14, 16, 31, 32]. Importantly, it is this final mechanism that is thought to contribute to PIs radiosensitizing effects. Inhibition of the PI3K/Akt pathway reduces vascular endothelial growth factor and hypoxia-inducible factor 1*α* which in turn leads to increased oxygenation and enhanced radiosensitivity [16]. This effect has been demonstrated in pre-clinical bladder [13], lung [13], and head and neck tumor xenografts [16] as well as clinical studies at a variety of sites including phase 1/2 non-small cell lung cancer [17, 18], phase I glioblastoma-multiforme [19], phase I cervical cancer [20, 21], phase I and II pancreatic cancer [22, 23], and phase I rectal cancer [24], which collectively have shown promising activity. Specifically regarding the phase I rectal cancer study, which represents an adjacent cancer site, concurrent use of the protease inhibitor nelfinavir with hypofractionated radiation therapy resulted in 5/9 patients achieving regression as well as increased blood perfusion as assessed by perfusion-CT [24].

Thus, while protease inhibitors have shown promising findings, fears that enhanced radiosensitization may result in increased toxicity and associated treatment complications persist. While this literature remains relatively novel, toxicity for combined radiotherapy-PI use has been found to be tolerable. One retrospective study of PLWH receiving radiotherapy for a variety of cancer sites found no difference in toxicity rates with or without PI use [33]. Additionally, the phase I-II studies discussed above cumulatively show overall tolerability of chemoradiotherapy with nelfinavir at the recommended FDA dose as an anti-retroviral agent in patients who are HIV negative in multiple tumor sites.

Although our study found increased hospitalizations due to acute hematologic toxicities in PLWH treated with PIs during chemoradiation, no other acute or chronic toxicity difference was found. It is important to take these results in the context of several factors. For one, it is necessary to note that the patients using PIs in our report were at greater baseline hematologic risk, as evidenced by the significantly greater proportion of patients with a CD4 count equal to or less than 200 at time of cancer diagnosis (28.1% vs 12.1%, *p* < 0.01). Secondly, PIs have also been found to potentiate myelotoxicity in some chemotherapy regimens due to alterations in the metabolism of chemotherapy agents via CYP3A4 enzyme inhibition [34]. And finally, the PI cohort had a higher degree of patients treated with the historically hemotoxic chemotherapy regimens that utilized mitomycin, 5-FU, and cisplatin, compared to the non-PI group. Thus, while this enhanced toxicity may be secondary to PI-based radiosensitization, there remains a multitude of factors to consider including potential drug interactions. Overall, it is vital to approach these results with the context that anal cancer treatment toxicity is often severe enough to prolong treatment duration and limit treatment completion, both of which have been observed to worsen overall outcomes [35–37]. In our study we found that PI use was not associated with prolonged duration of treatment, dose reduction of chemotherapy, or any change in radiation completion rate.

While the use of PIs was associated with an increased risk of hospitalization due to hematologic toxicity, and hospitalizations were associated with decreased OS, PI use itself was not associated with OS on either univariate or multivariable analysis. This may be due to the fact that while PIs increase hospitalizations due to neutropenia or lymphopenia, hospitalization may be attributable to a multitude of other factors that play a more significant role in OS. For example, hospitalizations due to hematologic toxicities are often easily treated (via granulocyte colony stimulating factors, or appropriate antibiotics for infections, etc.) than hospitalizations due to other treatment toxicities, such as radiation dermatitis, which may more substantially prolong or disrupt treatment. In addition, it may also be a cumulative effect of multiple toxicities that is needed to elicit those worsened outcomes rather than hematologic toxicities alone.

Another interesting finding was that patients with BMI between 25 and 30, or clinically overweight though non-obese, had improved OS compared to those with BMI < 25 or with normal BMI in multivariable Cox regression analysis. Likely, BMI within the overweight but non-obese range is a surrogate for higher performance status or better overall health of the patient at time of cancer diagnosis, as described in prior studies [38]. Performance status itself has been associated with cancer survival in multiple disease sites [39]. Unfortunately, performance status was not able to be assessed directly in this retrospective study.

There are multiple advantages of this study including the large diverse nationwide sample size of PWLH with anal cancer seen in the equal access VA healthcare system, and the availability of the specific ART medications that the patients were taking during treatment. We employed a rigorous multi-data source design including utilizing data from an adjudicated national cancer registry database as well as confirmatory chart review performed by clinical oncologist. We also used a rigorous tiered approach for multivariable analyses to balance maximizing study power and reliability of effect estimates for PI use with observed outcomes while also adjusting for key or strong confounders. Further, our sensitivity analysis of excluded patients who had changed their ART medications within 90 days of starting radiation treatment found no significant difference in any survival or recurrence outcome in Cox regression analysis and thus supports the internal validity and robustness of study findings. However, this study has several limitations. The major limitation of this study is its retrospective nature, which precludes causal inferences and the inability to independently confirm adherence of patients taking their ART medications given our medical records-based study design. Additionally, given the relative rarity of anal cancer, while higher among PLWH, we had sample size-related power considerations that necessitated limiting the number of potential confounders we could account for in multivariable analyses. In addition, all PIs were grouped together in this analysis. It is possible that certain generations of the drug, or even specific PIs, have different individual effects. However, due to the paucity of research in this area and the limited sample size, it was decided that grouping all PIs together was the best initial approach. An inherent confounder in our analysis is the role of HIV in the natural course of anal carcinoma. HIV-infection has been shown to result in increased recurrence rates and overall worsened outcomes [3–5]. We attempted to control for HIV-related variables including era during which patients were treated as well as CD4 count. Interestingly, nadir CD4 count was the only HIV-related variable retained in any multivariable model, and was significantly associated with CFS. No HIV-related variable was found to be correlated with either OS or RFS. Larger multi-institutional studies are needed to confirm this given our limited sample size and retrospective nature. Nevertheless, as HIV-infected individuals are disproportionately affected by anal cell carcinoma and a large number are already taking PIs, these results remain applicable to this population.

This study constitutes one of the first analyses of PI use during CRT treatment for anal cell carcinoma. While in our report there was an increase in hospitalizations due to febrile neutropenia and other hematologic toxicities in patients taking PIs, their use was not associated with treatment variables associated with reduced survival, such as ability to complete the entire course of radiation or chemotherapy dose reduction. Importantly, the use of PIs was not associated with changes in outcomes. Our study suggests that PLWH and anal cancer on PIs who are receiving chemotherapy and radiation may benefit from receiving prophylactic granylocyte colony stimulating factor and close monitoring for hematologic toxicities to prevent hospitalizations. Further prospective and randomized clinical research needs to be done examining the association between specific PIs and their radiation sensitizing effects in both immunocompetent and HIV-infected populations. In addition, further optimization is needed in the treatment of anal cancer to improve overall outcomes as well as to reduce both acute and long-term toxicities.

## Conclusions

The use of protease inhibitors during definitive CRT for the treatment of anal carcinoma in PLWH did not affect survival or recurrence outcomes. Their use was associated with increased hospitalizations due to hematologic toxicity, though not with any treatment variables associated with survival or recurrence. As HIV-infection continues to become a chronic disease, and the anal cancer disease burden increases, the treatment of anal cancer in PLWH will become more common. It is important for the medical community to understand the possible effects that drugs used to treat this disease, particularly potentially radiosensitizing drugs such as PIs, can have during radiation treatment.

## Data Availability

We utilized the Corporate Data Warehouse (CDW Database) accessed through the VA Informatics and Computing Infrastructure (VINCI) available to researchers in the VA community (VINCI Database). Consistent with policies of the U.S. Department of Veterans Affairs, this is a closed databased that is not available to the public; therefore, the authors do not have the ability to provide the dataset. To conduct this study, we received administrative permission to access and use these data in the Ethics approval and consent to participate section of our IRB as granted by National Data Systems.

## Abbreviations

PLWH: People living with HIV
ART: Antiretroviral therapy
CRT: Chemoradiation
NCCN: National Comprehensive Cancer Network
PI: Protease Inhibitor
VA: Department of Veterans Affairs
VINCI: VA Informatics and Computer Infrastructure
CDW: Corporate Data Warehouse
CCR: Central Cancer Registry
BMI: Body mass index
OS: Overall survival
RFS: Recurrence-free survival
CFS: Colostomy-free survival
PH: Proportional hazards
HR: Hazard ratio
CI: Confidence interval
IQR: Interquartile range
IMRT: Intensity-modulated radiation therapy
adj: Adjusted
OR: Odds ratio
